# Rewiring tumor metabolism: heterogeneity, oncometabolites, and adaptive plasticity

**DOI:** 10.1186/s40164-026-00795-z

**Published:** 2026-06-15

**Authors:** Jianqiang Yang, Catherine C. Zhou, Soumya Vijaya Kumar, Chloe Shay, Yong Teng

**Affiliations:** 1 Department of Hematology and Medical Oncology, Winship Cancer Institute, Emory University, Atlanta, GA 30322 USA; 2 Wallace H. Coulter Department of Biomedical Engineering, Georgia Institute of Technology and Emory University, Atlanta, GA 30322 USA

**Keywords:** Tumor metabolism, Metabolic heterogeneity, Metabolic reprogramming, Oncometabolites, The tumor microenvironment, Personalized treatment

## Abstract

Metabolic reprogramming is a well-established hallmark of cancer, driven by oncogenic mutations and microenvironmental pressures to support rapid proliferation and adaptation to stress. This reprogramming is not uniform but instead exhibits profound metabolic heterogeneity, with distinct tumor subpopulations and spatial niches relying on divergent metabolic pathways such as glycolysis, oxidative phosphorylation, and nutrient scavenging. This heterogeneity evolves dynamically during tumor progression and in response to therapy, driven by genetic and epigenetic alterations, hypoxia, nutrient gradients, and therapeutic influences. Critically, it contributes to major clinical challenges by promoting immune evasion through nutrient competition and the accumulation of immunosuppressive metabolites, and by fostering therapy-tolerant cell states. A deeper understanding of the spatiotemporal dynamics of tumor metabolism and their functional consequences reveals novel therapeutic vulnerabilities. This review examines the origins and biological implications of metabolic heterogeneity, discusses strategies to overcome therapeutic resistance by targeting context-specific metabolic dependencies or by combining metabolic inhibitors with immunotherapy, and highlights the potential of patient stratification based on metabolic profiles. Ultimately, elucidating this metabolic complexity is essential for the development of innovative, personalized therapeutic approaches that improve outcomes in cancer treatment.

## Introduction

Cancer cells frequently exhibit altered metabolic programs that support increased proliferation and survival while enabling adaptation to the complex tumor microenvironment (TME) [1, 2]. This phenomenon, termed metabolic reprogramming, encompasses coordinated changes in glucose, amino acid, and lipid metabolism that sustain rapid tumor growth, metastatic progression, and resistance to stress [3, 4]. Fundamentally, tumor metabolism refers to the ensemble of biochemical pathways and nutrient utilization strategies that cancer cells adopt to fulfill their unique bioenergetic, biosynthetic, and redox demands. It represents a profound divergence from the metabolic homeostasis maintained by normal, differentiated cells, shifting the cellular priority from efficient energy production to the rapid generation of biomass and the maintenance of survival under stress. Metabolic reprogramming is widely regarded as a hallmark of cancer [5]. By conferring metabolic plasticity, it allows tumor cells to dynamically adjust their metabolic fluxes in response to intrinsic oncogenic signals and extrinsic environmental challenges. Importantly, the pronounced heterogeneity of the TME, marked by steep spatial gradients in nutrient availability, oxygen tension, and stress signals, acts as a selective pressure that differentially shapes tumor metabolism. These spatially resolved adaptations can stabilize region-specific metabolic programs and thereby contribute to intratumoral metabolic heterogeneity [6, 7].

A critical but often underappreciated question is whether metabolic heterogeneity arises merely as a passive consequence of microenvironmental gradients or instead reflects an active and adaptive program that tumors exploit to evade therapy and immune surveillance. This distinction has major therapeutic implications. If metabolic heterogeneity is largely passive and spatially constrained, broad metabolic targeting strategies may be sufficient to suppress tumor growth. Conversely, if tumor cells can dynamically and reversibly transition between metabolic states, static pathway inhibition is unlikely to produce durable responses, and effective therapies will need to disrupt the mechanisms governing metabolic plasticity and state switching itself [8, 9]. Importantly, definitive in vivo evidence demonstrating active and reversible metabolic state switching in human tumors remains limited. Much of the existing literature relies on static molecular snapshots or short-term in vitro perturbation models, which may inadequately capture the temporal dynamics and environmental complexity of the human TME. Consequently, current enthusiasm surrounding “state-switching” therapeutic strategies should be tempered by the recognition that longitudinal, high-resolution metabolic monitoring in patients is still lacking. Further advances in real-time metabolic imaging and lineage-resolved profiling will be essential to determine whether metabolic plasticity represents a true driver of therapeutic resistance in vivo or primarily reflects context-dependent adaptation.

Normal cells flexibly balance nutrients used to support tissue homeostasis, whereas cancer cells rewire metabolism, favoring aerobic glycolysis, heightened glutamine and lipid utilization, and stress-adaptive pathways, to sustain rapid proliferation and survival in hostile microenvironments (Table 1). In normal tissues, cellular metabolism is tightly regulated to match energetic demand with nutrient supply, prioritizing efficient energy production via oxidative phosphorylation (OXPHOS) and maintaining physiological functions. Normal cells primarily generate ATP through mitochondrial respiration, utilizing metabolic checkpoints like the AMP-activated protein kinase (AMPK) pathway to couple catabolism with anabolism [10, 11]. Differentiation status and tissue-specific functions dictate distinct but stable metabolic phenotypes, such as lipogenesis in the liver or fatty acid oxidation in cardiac muscle, all operating within a framework of systemic hormonal control [12, 13]. In contrast, cancer cells hijack normal metabolic control; fueled by oncogenic alterations and loss of tumor suppressors, they reprogram metabolism toward rapid biomass production at the expense of energetic efficiency. The most iconic feature is the Warburg effect or aerobic glycolysis, wherein cancer cells avidly take up glucose and convert it to lactate even in the presence of ample oxygen [2]. While yielding fewer ATP molecules per glucose, glycolysis provides a rapid ATP turnover and, critically, floods the cell with metabolic intermediates (e.g., glucose-6-phosphate, 3-phosphoglycerate) that serve as building blocks for nucleotides, amino acids, and lipids. This shift from an energy-efficient to a biosynthesis-prioritized state is further enabled by concurrent dependencies on glutamine for nitrogen and anaplerosis, and on de novo lipogenesis for membrane production [14, 15]. Unlike normal cells, cancer cells exhibit constitutive, oncogene-driven expression of nutrient transporters (e.g., GLUT1, ASCT2) and lose the metabolic brakes imposed by tumor suppressors like p53, which normally promotes OXPHOS and suppresses glycolysis [16–18]. This reprogramming is not a passive consequence but a dynamic, enabling trait that integrates intrinsic oncogenic signals with extrinsic environmental pressures, supporting all hallmarks of cancer and creating a self-reinforcing loop that sustains the tumor ecosystem [2, 19].

Distinct cancer cell subpopulations exhibit different metabolic preferences. It has been reported that hypoxic cancer regions are often glycolysis-dominant, while oxygenated tumor prefers OXPHOS [20]. Intra-tumoral heterogeneity (ITH) can exist either between geographical areas of the same tumor (spatial heterogeneity) or between different lesions that appear over time locally or distantly (temporal heterogeneity) [21]. In addition, specialized subgroups, such as cancer stem cells (CSCs) and circulating tumor cells (CTCs), exhibit unique dependencies on signaling pathways, such as fatty acid oxidation or glutaminolysis, and eventually enhance survival during stress and metastasis [20]. At the intertumoral level, cancers from different tissues or with distinct oncogenic drivers display unique metabolic phenotypes. For example, *KRAS*-mutant pancreatic tumors rely heavily on amino acid scavenging, whereas glioblastomas predominantly exhibit reprogrammed lipid metabolism [22–24]. These differences highlight how tissue context and genetic background shape metabolic identity. The TME further contributes by introducing metabolic heterogeneity across non-tumor components. Stromal cells, such as cancer-associated fibroblasts (CAFs), adipocytes, and tumor-infiltrating immune cells (TIICs), undergo their own metabolic rewiring and participate in nutrient competition and metabolite exchange with cancer cells. This metabolic crosstalk not only supports tumor progression but also shapes immune responses within the TME [20].

While the major pathways of metabolic reprogramming are well established, this review highlights several emerging concepts that remain underappreciated. First, we frame intratumoral metabolic heterogeneity not merely as a passive consequence of microenvironmental gradients, but as a dynamic and adaptive survival program that tumors may exploit to evade therapy and immune attack. This perspective positions metabolic heterogeneity as a driver of tumor adaptation and resistance rather than a static feature of tumor architecture. We also emphasize the context dependency of oncogene-driven metabolism. Identical genetic alterations can produce distinct, or even opposing, metabolic phenotypes depending on tissue lineage and cellular context. For example, combined *KRAS*^*G12D*^ activation and *TP53* loss enhances branched-chain amino acid (BCAA) utilization in non-small cell lung cancer (NSCLC) but suppresses BCAA metabolism in pancreatic ductal adenocarcinoma (PDAC), challenging the assumption that genotype alone determines metabolic dependency. In addition, we synthesize the mixed clinical outcomes of metabolic therapies. Although mutant IDH inhibitors have achieved clinical success, glycolytic inhibitors have shown limited efficacy, and metformin has produced inconsistent results across tumor types. To address these challenges, we discuss emerging combination strategies that simultaneously target primary metabolic vulnerabilities and the compensatory pathways activated during treatment. Collectively, this review provides an integrated framework for understanding how metabolic heterogeneity and plasticity shape tumor progression, therapeutic resistance, and treatment response.

**Table 1:** Comparative metabolic profile of normal and cancer cells

Metabolic profile	Normal cells	Cancer cells
Primary energy pathway	Primarily rely on oxidative phosphorylation, generating ATP efficiently (approximately 30–36 ATP per glucose molecule) under aerobic conditions.	Prefer aerobic glycolysis (Warburg effect), producing lactate and substantially less ATP (approximately 2 ATP per glucose molecule) even in the presence of oxygen [25].
Nutrient utilization	Glucose: uptake is tightly regulated according to cellular demand (e.g., GLUT4-mediated transport). Glutamine: functions as a nitrogen donor for biosynthesis and contributes to redox balance. Lipids: fatty acid uptake, synthesis, storage, and catabolism are tightly controlled to maintain metabolic homeostasis.	Glucose: frequently exhibit elevated glucose uptake through transporters such as GLUT1 [25]. Glutamine: often become glutamine-dependent, using glutamine as a major carbon and nitrogen source to sustain proliferation, redox homeostasis, and survival [26, 27]. Lipids: enhanced fatty acid uptake and de novo lipid synthesis support membrane production, energy storage, and signaling [28].
Mitochondrial role	Serve primarily as the cellular powerhouse, producing most ATP through oxidative phosphorylation while also regulating calcium homeostasis, apoptosis, and biosynthetic processes.	Function as multifunctional metabolic hubs involved in metabolic rewiring, oncometabolite generation, immune evasion, redox regulation, and therapeutic resistance [29, 30].
Metabolic priorities	Maintain energy efficiency, cellular homeostasis, and tissue-specific physiological functions.	Prioritize rapid proliferation and survival by supporting biomass production, including nucleotides, proteins, and lipids, alongside energy generation [30, 31].
Key regulatory drivers	Controlled mainly by physiological cues, including hormones (e.g., insulin), nutrient-sensing pathways (AMPK, mTOR), and oxygen availability.	Driven by oncogenic signaling pathways, including MYC, RAS, and AKT activation, as well as loss of tumor suppressors such as p53, leading to sustained anabolic signaling [30, 32].
Response to hypoxia/anoxia	Hypoxia transiently induces glycolysis through HIF-1α activation and may trigger cell-cycle arrest or apoptosis under severe stress.	Persistent HIF-1α activation promotes glycolysis, angiogenesis, invasion, and resistance to cell death under hypoxic conditions [33, 34].
Impact on the microenvironment	Maintain tissue homeostasis, with metabolic by-products such as reactive oxygen species (ROS) remaining within physiological ranges.	Remodel TME through secretion of metabolites such as lactate, which suppress immune cell function and promote invasion and metastasis [25, 29].

## Sources of metabolic heterogeneity

**Fig. 1:**
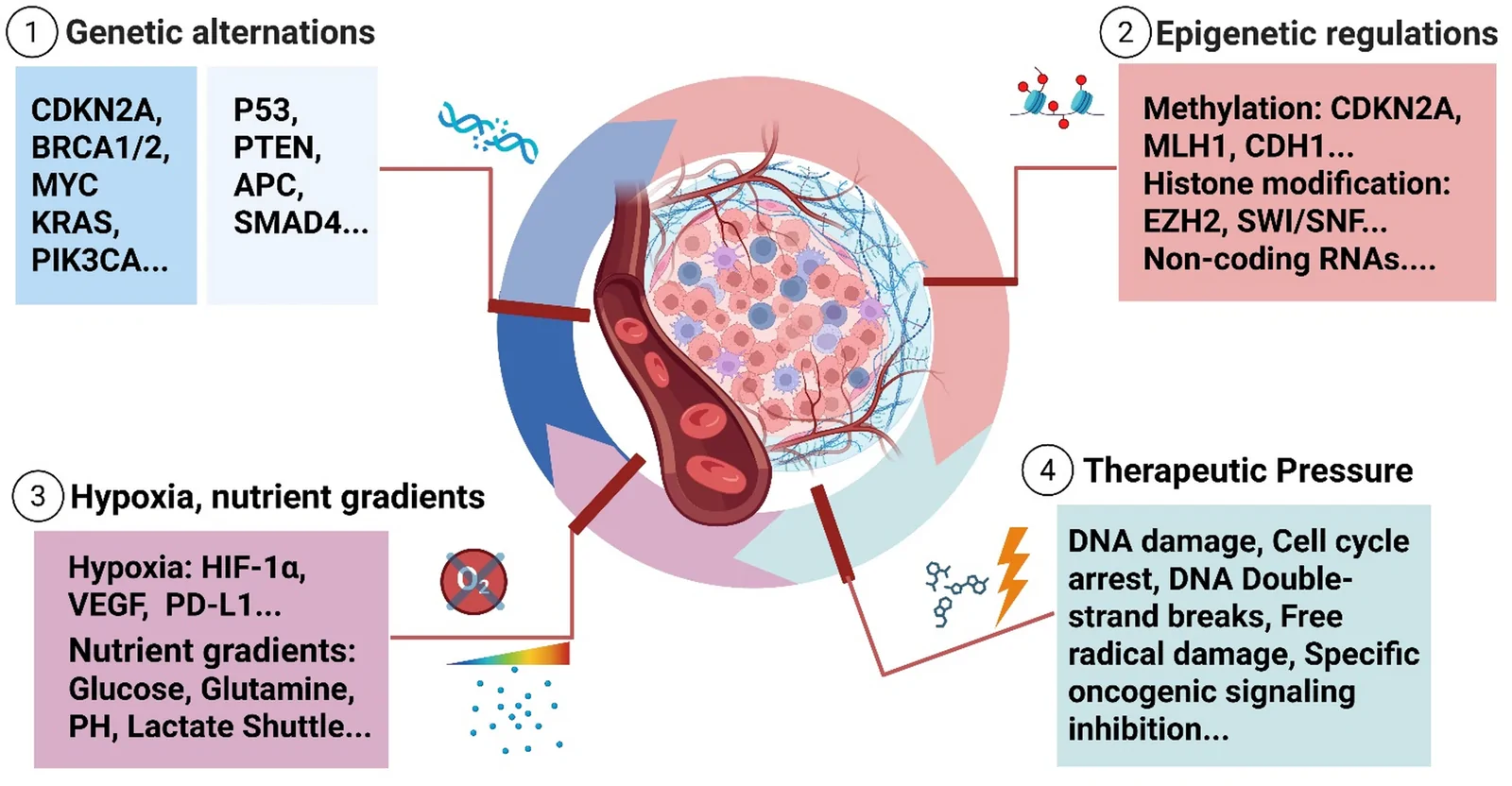
Sources of tumor metabolic heterogeneity. The schematic summarizes the major drivers of intratumoral metabolic diversity, which originate from four interconnected domains: (1) genetic alterations (e.g., oncogene activation, tumor suppressor loss), (2) epigenetic regulation (e.g., DNA methylation, histone modifications), (3) microenvironmental gradients (e.g., hypoxia, nutrient availability), and (4) therapeutic pressure. The interplay of these factors generates a mosaic of metabolic phenotypes that promote tumor adaptation, disease progression, and therapy resistance. Figure created using BioRender (https://biorender.com)

Metabolic heterogeneity in cancer is a multifactorial phenomenon, manifesting at the inter-patient, inter-tumor, and intra-tumor levels. It is driven by the interplay of cell-autonomous factors, such as oncogenic mutations, and non-cell-autonomous cues from the TME. Metabolic heterogeneity arises from differences in genetic mutations, epigenetic regulation, and gene expression that alter metabolic enzyme activity across cells (Fig. 1). It is also shaped by microenvironmental factors such as nutrient availability, oxygen levels, and signaling interactions, which force cells to adapt their metabolic pathways (Fig. 1). Additionally, developmental stage, cellular differentiation, and therapeutic pressure contribute to distinct metabolic states within the same tissue (Fig. 1).

### Genetic and epigenetic drivers

Metabolic heterogeneity is driven by the concerted action of mutated oncogenes, inactivated tumor suppressors, and epigenetic alterations. These layers of regulation systematically reprogram the fluxes of glucose, amino acids, and lipids to meet the biosynthetic and energetic demands of rapid proliferation (Fig. 1).

The MYC oncogene functions as a master transcriptional amplifier of anabolic metabolism. It directly upregulates genes involved in glycolysis, including glucose transporters (e.g., GLUT1) and key glycolytic enzymes. Concurrently, MYC promotes glutaminolysis by inducing the expression of glutamine transporters (SLC38A5 and SLC1A5) and glutaminase (GLS1), thereby supplying nitrogen and carbon for nucleotide and lipid biosynthesis [27, 35]. Building on this, our previous study demonstrated that MYC and GLS1 form a positive feedback loop that further enhances glutaminolysis, driving head and neck cancer metastasis. Importantly, disruption of this loop suppresses glutaminolysis and effectively inhibits the progression of head and neck cancer [36]. Furthermore, MYC stimulates *de novo* lipid synthesis to support membrane biogenesis. These broad programs create a state of oncogene addiction, where tumor survival becomes exquisitely dependent on MYC activity, as demonstrated by dramatic tumor regression upon its inactivation in preclinical models [17]. Similarly, *KRAS* mutations, which are ubiquitous in cancers such as PDAC, reprogram cellular metabolism through hyperactivation of downstream MAPK signaling. *KRAS*-driven tumors exhibit increased glucose uptake and glycolytic flux, largely mediated by ERK activation. In parallel, they depend on enhanced and often noncanonical glutamine metabolism to maintain redox homeostasis, while sustaining rapid biomass accumulation through de novo lipogenesis and nutrient-scavenging processes such as micropinocytosis [37].

In contrast to oncogenic drivers, the tumor suppressor p53 functions as a key regulator of metabolic homeostasis. It counterbalances anabolic metabolism by repressing glycolysis through downregulation of glucose transporters (GLUT1/4/12) and glycolytic enzymes such as hexokinase 2 (HK2), while simultaneously promoting mitochondrial oxidative phosphorylation. p53 mediates these effects by activating pyruvate dehydrogenase and inducing the transcription of respiratory chain components, including SCO2 [37, 38]. In addition, p53 fine-tunes amino acid metabolism by inducing glutaminase 2 (GLS2), thereby linking glutamine utilization to energy production and the regulation of ferroptosis [38]. Additionally, p53 constrains lipogenesis via repression of SREBP-1c and enhances fatty acid oxidation. Its loss, therefore, removes a critical brake on metabolic flexibility, facilitating the adaptive and aggressive metabolism characteristic of cancer cells [38, 39]. A hallmark example is oncogenic *KRAS*, which is mutated in the majority of PDAC. *KRAS* reprograms glucose metabolism by enhancing glycolysis and redirecting glutamine through a non-canonical pathway. Unlike normal cells, which primarily rely on mitochondrial GLUD1 to convert glutamine into α-ketoglutarate (α-KG) for entry into the tricarboxylic acid (TCA) cycle, PDAC cells reroute glutamine through a distinct cytoplasmic pathway. In this pathway, glutamine-derived aspartate is sequentially converted to oxaloacetate, malate, and ultimately pyruvate, leading to an increased NADPH/NADP⁺ ratio that sustains redox homeostasis and reduced glutathione levels, thereby protecting PDAC cells from oxidative stress [40]. Notably, identical oncogenic mutations can drive divergent metabolic programs depending on tissue context: whereas *KRAS*^*G12D*^ combined with TP53 loss enhances BCAA uptake and catabolism in NSCLC, the same genotype suppresses BCAA utilization in PDAC, highlighting how clonal drivers interact with non-clonal factors to generate distinct metabolic dependencies in solid tumors [41].

Beyond genetic alterations, epigenetic regulators such as the SWI/SNF complex introduce an additional layer of metabolic complexity. Rather than generating fixed metabolic programs, SWI/SNF mutations often disrupt canonical metabolic regulation, forcing tumor cells to rely on compensatory pathways, such as oxidative phosphorylation, that may subsequently become therapeutic vulnerabilities [42]. Importantly, identical SWI/SNF alterations can drive different metabolic phenotypes depending on tissue context. For example, knockdown of SMARCA4 (also known as BRG1), a catalytic subunit of SWI/SNF, in breast cancer cells suppresses de novo lipid synthesis and reduces proliferation, while in lung cancer cells, SMARCA4 acts as a potent tumor suppressor and its loss forces a profound metabolic shift, making tumor cells highly dependent on OXPHOS [43, 44]. These observations underscore that genotype alone is insufficient to predict metabolic behavior; tissue lineage and microenvironmental signals are equally critical determinants. Consequently, effective metabolic biomarkers for patient stratification will likely require integrated genetic, epigenetic, and spatial profiling rather than reliance on mutation status alone.

### Hypoxia and nutrient gradients

Hypoxia and nutrient gradients within the TME impose strong selective pressures that drive region-specific metabolic adaptations in cancer cells (Fig. 1). These spatially organized responses promote the establishment of distinct metabolic states across different tumor regions and contribute to the development and maintenance of intratumoral metabolic heterogeneity.

A defining feature of solid tumors is hypoxia, resulting from disorganized, leaky vasculature unable to support rapid tumor growth. In hypoxic regions, hypoxia-inducible factors (HIF-1α and HIF-2α) are stabilized and activate transcriptional programs that promote survival and aggression [45]. HIFs drive angiogenesis (e.g., via VEGF) [46], induce a metabolic shift toward glycolysis (via LDHA) [47], and inhibit mitochondrial oxidation (via PDK1) [48]. Functionally, hypoxia confers resistance by impairing radiotherapy (which requires oxygen as a radiosensitizer), rewiring DNA repair pathways, and upregulating drug efflux pumps. Furthermore, it fosters immune evasion by upregulating checkpoint ligands like PD-L1 and recruiting immunosuppressive cells such as myeloid-derived suppressor cells (MDSCs), establishing a resilient tumor niche.

Concurrent with oxygen depletion, chaotic vasculature and high metabolic demand create profound nutrient gradients, leading to the depletion of glucose, glutamine, and amino acids within tumor cores [49]. Cancer cells adapt by overexpressing nutrient transporters (e.g., GLUT1, ASCT2), activating scavenging pathways like macropinocytosis, and engaging in metabolic symbiosis with stromal cells. A canonical example is pancreatic cancer cells importing alanine secreted by cancer-associated fibroblasts to sustain the TCA cycle under glucose-limiting conditions [50]. Such metabolic adaptations promote survival under stress and select for aggressive clones with heightened metabolic flexibility, thereby driving intratumoral heterogeneity and conferring resistance to therapies that target individual metabolic pathways. Moreover, the high glycolytic flux in hypoxic regions, coupled with the activity of carbonic anhydrases (e.g., CAIX, CAXII), leads to massive lactate secretion and the development of extracellular acidosis (tumor pH ~ 6.5–6.9 vs. normal tissue pH ~ 7.4) [51, 52]. This acidic milieu is a potent immunosuppressor, directly impairing the function of cytotoxic T cells and NK cells. Additionally, acidosis can alter the charge and structure of chemotherapeutic agents, reducing their cellular uptake and efficacy [53, 54].

### Oncometabolites

A distinctive and therapeutically important dimension of metabolic heterogeneity arises from oncometabolites, metabolites that accumulate to supraphysiological levels due to mutations in metabolic enzymes and function as oncogenic signaling molecules that reshape epigenetic and cellular states [55, 56]. The best-characterized example is D-2-hydroxyglutarate (D-2-HG), generated through the neomorphic activity of mutant IDH1 or IDH2. D-2-HG competitively inhibits α-ketoglutarate (α-KG)-dependent dioxygenases, including TET DNA demethylases and JmjC-domain histone demethylases, leading to widespread DNA and histone hypermethylation. This epigenetic rewiring promotes a stem-like, differentiation-blocked cellular state [57, 58]. Importantly, intratumoral variability in IDH mutation status or regional differences in D-2-HG production can generate metabolically and epigenetically distinct tumor subpopulations [59]. D-2-HG also exerts non-cell-autonomous effects by impairing mitochondrial respiration and glycolysis in infiltrating CD8^+^ T cells while promoting regulatory T cell differentiation, contributing to an immunosuppressive microenvironment [60, 61].

Similar mechanisms occur in tumors with fumarate hydratase (FH) or succinate dehydrogenase (SDH) deficiency. Accumulation of fumarate or succinate inhibits α-KG-dependent dioxygenases, stabilizes HIF-1α, and disrupts histone and DNA demethylation, resulting in pseudohypoxia and epigenetic silencing of tumor-suppressor programs [62, 63]. Spatial variation in these metabolites within tumors may further create regional differences in hypoxic signaling, differentiation state, and epigenetic regulation, embedding metabolic heterogeneity directly into tumor architecture. Notably, oncometabolite-like functions are not restricted to rare metabolic enzyme mutations. Lactate, which accumulates broadly in hypoxic tumors, can stabilize HIF-1α through inhibition of PHD2, serve as a substrate for histone lactylation, and suppress antitumor immunity by impairing NK-cell and dendritic-cell function [64, 65]. Thus, metabolites can simultaneously function as energy substrates, epigenetic regulators, and intercellular signaling molecules, with their effects determined by local concentration, compartmentalization, and cellular context.

Given that oncometabolites directly alter the enzymes that regulate epigenetic modifications, even transient or spatially restricted metabolic changes may become epigenetically stabilized as heritable transcriptional states, thereby reinforcing phenotypic heterogeneity over time. This concept is exemplified by the clinical success of FDA-approved IDH inhibitors such as ivosidenib and enasidenib, which reverse D-2-HG-driven epigenetic dysregulation and restore differentiation in IDH-mutant malignancies [66, 67]. However, such genetically defined dependencies are relatively uncommon in solid tumors. More frequently, oncometabolite-like effects arise from abundant and context-dependent metabolites, including lactate, succinate, and reactive oxygen species, whose redundancy and compensatory interactions may require combinatorial therapeutic strategies. Importantly, comprehensive mapping of oncometabolite landscapes across cancers remains incomplete, as most current knowledge derives from IDH-, FH-, or SDH-mutant models, highlighting the need for integrated metabolomic and epigenomic profiling in broader patient populations.

### Therapeutic pressure

Therapeutic interventions represent a potent extrinsic selective pressure that actively shapes and amplifies metabolic heterogeneity within tumors (Fig. 1). Treatment-induced metabolic adaptations are a key driver of both intrinsic and acquired resistance, introducing a temporal heterogeneity that evolves throughout tumor progression [68, 69].

The acute metabolic response to therapy involves rapid, often reversible, rewiring of cellular metabolism to withstand cytotoxic stress. For example, chemotherapy and radiotherapy commonly induce oxidative stress, prompting cancer cells to upregulate antioxidant synthesis pathways, such as glutathione production fueled by glutaminolysis [70, 71]. Inhibition of dominant oncogenic signaling pathways like PI3K/AKT/mTOR can trigger immediate compensatory metabolic shifts, including increased mitochondrial OXPHOS and enhanced dependence on alternative nutrients such as fatty acids or amino acids, enabling tumor cells to bypass the targeted blockade [72, 73]. A critical consequence of therapeutic pressure is the selection and stabilization of drug-tolerant persister (DTP) cells. These cells adopt a slow-cycling or quiescent state marked by extensive epigenetic remodeling and a profound metabolic reprogramming [74]. This reprogramming typically involves downregulation of glycolysis and a shift toward increased reliance on mitochondrial respiration, often accompanied by elevated fatty acid oxidation [75]. This altered metabolic phenotype reduces biosynthetic activity and diminishes the effectiveness of therapies designed to target rapidly proliferating cells, thereby allowing DTPs to survive treatment and act as a reservoir for eventual relapse and clonal evolution [76, 77]. Moreover, sustained therapeutic pressure drives Darwinian selection of subclones harboring preexisting or acquired genetic alterations that confer metabolic advantages under drug exposure [78]. This selective process promotes the expansion of cell populations with distinct and stable metabolic dependencies, including enhanced nutrient scavenging via macropinocytosis or increased reliance on specific amino acids, which deviate from the metabolic profile of the pretreatment tumor bulk [79, 80]. Consequently, therapy not only selects for resistant clones but actively reshapes the tumor’s metabolic landscape, generating a therapy-adapted heterogeneous state that poses a substantial barrier to durable remission [81].

## Spatial-temporal metabolic heterogeneity

**Fig. 2:**
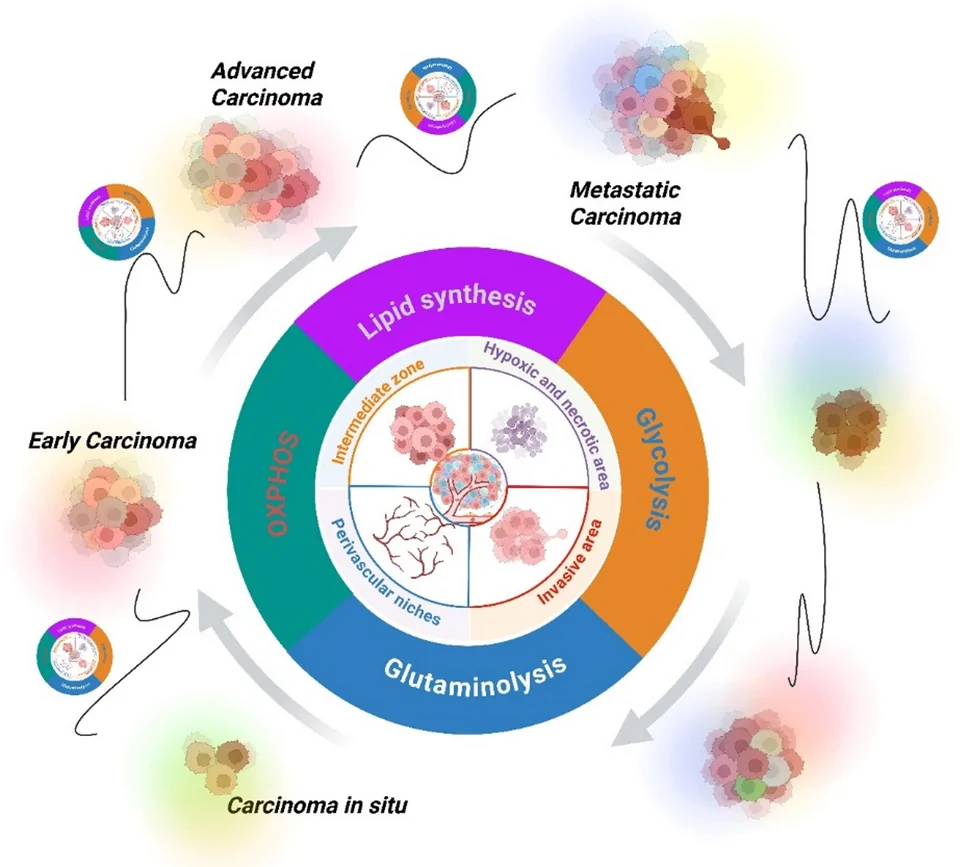
Spatiotemporal evolution of intratumoral metabolic programs. The outer ring depicts the temporal progression from carcinoma in situ to metastatic cancer. The concentric inner diagram represents a tumor cross-section at each stage. The inner ring denotes four spatial niches, perivascular, intermediate, invasive front, and hypoxic/necrotic core, while the outer ring shows four core metabolic programs: glycolysis, oxidative phosphorylation (OXPHOS), glutaminolysis, and lipid synthesis. During tumor progression, genetic alterations and microenvironmental gradients dynamically reshape the dominant metabolic pathways within each spatial compartment, illustrating that metabolic heterogeneity is both spatially organized and temporally plastic

Spatial and temporal metabolic heterogeneity are not independent processes, but interconnected dimensions of tumor adaptation. Temporal metabolic shifts driven by oncogenic evolution, microenvironmental changes, or therapeutic pressure can progressively reshape the spatial distribution of metabolic niches within tumors. Conversely, the pre-existing spatial architecture of the TME, particularly oxygen and nutrient gradients, constrains which metabolic programs can emerge and persist over time, thereby guiding region-specific evolutionary trajectories. As a result, spatial heterogeneity observed at any single time point represents only a snapshot of a continuously evolving metabolic landscape (Fig. 2). The following sections examine the spatial organization and temporal evolution of tumor metabolism while emphasizing the reciprocal interplay between these processes.

### Spatial metabolic heterogeneity

**Fig. 3:**
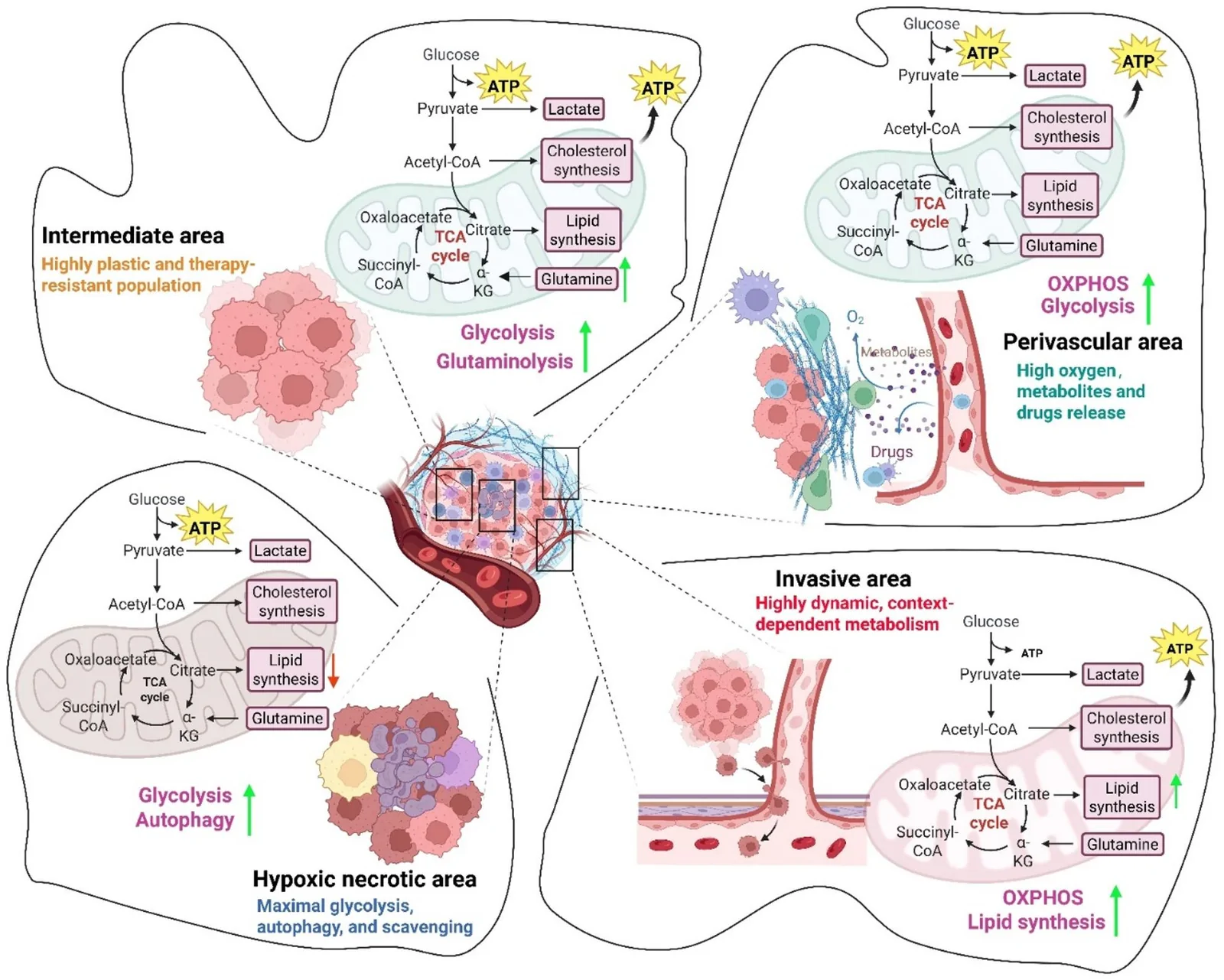
Spatial metabolic compartmentalization within the TME. The schematic illustrates the spatial organization of metabolic phenotypes within a solid tumor, shaped by gradients of oxygen, nutrients, and therapeutic pressure. Distinct functional niches emerge: the perivascular area, with abundant oxygen and drug access, utilizes both OXPHOS and glycolysis; the intermediate zone harbors highly plastic, therapy-resistant cells; the hypoxic and necrotic core relies predominantly on glycolysis and autophagy; and the invasive front exhibits a dynamic, context-dependent metabolism favoring lipid synthesis and OXPHOS. This metabolic compartmentalization plays a central role in promoting treatment resistance and tumor progression. Figure created using BioRender (https://biorender.com)

Spatial metabolic heterogeneity arises from localized differences in oxygen, nutrient availability, and stromal-immune interactions within the TME, driving region-specific metabolic programs (Fig. 3). In many solid tumors, these spatially distinct metabolic adaptations promote tumor progression, therapeutic resistance, and immune evasion, highlighting metabolic heterogeneity as a fundamental feature of cancer biology. However, the precise spatial organization of these metabolic states varies considerably across tumor types and has thus far been characterized in only a limited number of cancer models [68, 82]. Importantly, these spatial metabolic compartments are not static. As tumors progress and are exposed to therapeutic or environmental pressures, the metabolic identity of each niche can evolve over time, and tumor cells may dynamically transition between distinct metabolic regions.

#### Perivascular area

In well vascularized tumors, such as glioblastoma and certain lung cancers, the perivascular area, defined by its proximity to functional blood vessels, represents the most resource-rich niche within the tumor ecosystem. Far from being a passive recipient of nutrients, this area acts as a dynamic, metabolically dominant hub that sustains tumor biomass, shapes the systemic metabolic landscape, and mediates potent cell-extrinsic immune suppression through direct nutrient competition. Cancer cells in this niche often have immediate access to oxygen and nutrients, enabling robust aerobic glycolysis and OXPHOS for maximal ATP production, alongside high glutaminolysis and *de novo* lipogenesis to generate nucleotides, amino acids, and lipids for rapid proliferation [83–85]. The balance between glycolysis and OXPHOS in the perivascular niche is highly context-dependent and varies across tumor types and oncogenic drivers. For example, in *MYC*-driven tumors, perivascular cancer cells often depend strongly on glutaminolysis, whereas in *KRAS*-mutant pancreatic cancers, perivascular populations are frequently characterized by enhanced macropinocytosis that supports lipid acquisition and *de novo* lipid synthesis [50, 86, 87]. Elevated expression of transporters, such as GLUT1, SLC1A5/ASCT2, and LAT1 allows these cells to establish steep nutrient gradients, actively scavenging resources from the bloodstream before they diffuse into the tumor parenchyma [82, 88]. In certain tumor models, the perivascular area serves as a primary site of nutrient-mediated immune suppression, where cancer cells induce local pseudo-nutrient depletion that diminishes the metabolic fitness and effector function of infiltrating immune cells, leading to anergy or exhaustion before effective immune engagement [89, 90].

Located adjacent to blood vessels, cancer cells in the perivascular area often act as the first line of defense against drugs and metabolites. They employ sophisticated, active strategies not only to withstand but also to manipulate drug exposure and metabolite exchange, ensuring survival and promoting resistance. High proliferative activity elevates interstitial fluid pressure, hindering drug penetration into deeper tumor regions [91]. Concurrently, upregulation of efflux transporters such as P-glycoprotein (MDR1) actively expels chemotherapeutic agents [92], while overexpression of MCT4 facilitates lactate export, preventing intracellular acidification. This lactate-driven acidification of the extracellular environment impairs immune cell function and diminishes the effectiveness of chemotherapies [93]. The relative contribution of these resistance mechanisms is highly context-dependent, varying both across tumor types and among distinct perivascular regions within the same tumor. Moreover, the metabolic programs of perivascular cells are temporally dynamic. Changes in vascular function, including transient vascular normalization or exposure to anti-angiogenic therapy, can alter local nutrient and oxygen availability, thereby reshaping the metabolic state of this niche over time.

#### Intermediate area

In tumors with a classic zonal architecture, such as breast cancer and head and neck cancer, cancer cells in the intermediate region, located farther from blood vessels, experience intermittent hypoxia and fluctuating nutrient availability. To survive these conditions, they exhibit high metabolic plasticity, engaging both glycolytic and mitochondrial pathways. These cells primarily rely on glycolysis to cope with variable oxygen levels but maintain partial mitochondrial function, distinguishing them from the fully glycolytic hypoxic core. However, the extent of mitochondrial activity within this zone is tumor-type dependent. In some cancers, there is a well-defined metabolic boundary, with a sharp transition between oxidative, perivascular regions and more glycolytic, hypoxic tumor regions, whereas in others this gradient is more diffuse, reflecting greater metabolic plasticity across compartments. A key regulator in this region is HIF-1α, which remains stabilized even under intermittent hypoxia and drives the expression of glycolytic enzymes, lactate transporters, and immunosuppressive factors such as IDO1 and CD73 [47, 94, 95].

The adaptive features of cancer cells in the intermediate region contribute to resistance against multimodal therapies. Glutamine serves as a primary nitrogen and carbon source for glutathione (GSH) synthesis, the cell’s central antioxidant. The active glutaminolysis pathway continuously replenishes the GSH pool, allowing cells to efficiently neutralize reactive oxygen species generated by radiotherapy, certain chemotherapies (e.g., platinum agents), or intrinsic metabolic stress [27, 96]. This creates a direct biochemical barrier that protects against oxidative damage–induced cell death.

#### Invasive area

In many tumors, including breast, pancreatic, and colorectal cancer, the invasive front represents a critical and highly dynamic spatial niche in which cancer cells detach from the primary tumor mass and initiate local invasion as well as metastatic dissemination. Unlike the relatively stable architecture of the tumor core, this region is characterized by continuous interactions with the host stroma, including fibroblasts, immune cells, and a remodeled extracellular matrix (ECM) [97]. Tumor-associated mesenchymal stem/stromal cells actively participate in this bidirectional crosstalk, promoting carcinogenesis and remodeling the stromal niche to facilitate tumor invasion [98]. Concurrently, dynamic remodeling of the ECM not only imposes physical constraints on tumor expansion but also regulates immune surveillance by shaping immune cell infiltration and function [99]. In addition, increased ECM stiffness enhances tumor progression through activation of mechanotransduction pathways and has emerged as a potential therapeutic vulnerability [100]. More broadly, mesenchymal stromal cells within the TME contribute to tumor initiation, metastatic dissemination, and the establishment of an immunosuppressive niche [101]. The metabolic profile of cells in this niche differs from both the proliferative perivascular region and the hypoxic core, being specifically adapted to support cell motility, environmental adaptation, and immune evasion. However, metabolic adaptations at the invasive margin are not uniform across tumor types. For instance, certain sarcoma subtypes do not exhibit the same lipid-dependent invasive programs observed in epithelial cancers, underscoring substantial context dependency in invasive-edge metabolism [102]. Cancer cells in the invasive area operate under distinct physical and mechanical constraints that further shape their metabolic programs [103].

In epithelial cancers, invasive tumor cells often engage both glycolysis to rapidly generate ATP and OXPHOS to sustain energy-demanding migration, with many mesenchymal-like states showing a particular dependence on fatty acid–supported OXPHOS. However, the relative contribution of these metabolic pathways is highly context-dependent and varies with tumor tissue of origin and underlying genetic alterations [82], particularly in those with a mesenchymal phenotype. They import metabolites to fuel the mitochondrial TCA cycle and OXPHOS, supporting the energy-intensive processes required for invasion [68]. Lipid metabolism is also critical in this niche as cells require phospholipids for constructing new membranes, and *de novo* synthesized fatty acids can alter membrane composition and fluidity, facilitating motility and dynamic reshaping during migration [104, 105]. Beyond structural roles, lipids such as lysophosphatidic acid (LPA) and prostaglandins (e.g., PGE2) serve as signaling molecules that enhance migration and tumor–stroma interactions, while lipogenesis contributes to epithelial-mesenchymal transition (EMT), a key driver of invasion [106, 107]. Cancer cells import exogenous fatty acids via transporters like CD36 and FABP4, which are stored in lipid droplets or oxidized for energy to support invasion and metastatic survival. For example, co-culture of breast cancer cells with adipocytes upregulates CD36-mediated fatty acid uptake, followed by FABP4-mediated intracellular transport, which activates STAT3 signaling to promote EMT and stemness [108].

#### Hypoxic and necrotic area

In the severely hypoxic tumor core, cancer cells in many solid tumors are largely constrained to glycolysis for ATP production due to limited oxygen availability. However, this metabolic state is not absolute. Certain tumor cells, such as those with elevated HIF-2α activity or loss of p53, maintain residual oxidative metabolism even under hypoxic conditions. Accordingly, the extent of glycolytic dependence varies across tumor types and is shaped by their distinct genetic and regulatory contexts. This results in massive accumulation and secretion of lactic acid, causing pronounced extracellular acidification (pH 6.0-6.5). Additionally, cells adjacent to necrotic debris often engage in macropinocytosis to scavenge macromolecules, providing an alternative source of nutrients to sustain survival under extreme metabolic stress.

The acidic, lactate-rich environment of the tumor core is highly immunosuppressive. Low pH directly impairs T and NK cell cytotoxicity and inhibits lymphocyte proliferation, while lactate functions as a signaling molecule that stabilizes HIF-1α in immune cells, drives M2-like polarization of tumor-associated macrophages (TAMs), and upregulates PD-L1 expression across various cell types [109]. Coupled with poor drug penetration and low proliferative activity, the hypoxic tumor core can function as a protective niche that supports treatment persistence and resistance to systemic therapies. However, it remains unresolved whether necrotic regions actively contribute to immunosuppression through released metabolites (e.g., ATP-to-adenosine signaling) or instead represent passive by-products of tumor growth; clarifying this distinction will be essential for determining whether necrosis itself is a viable therapeutic target.

Together, these spatially defined regions form a continuous metabolic gradient rather than isolated compartments, collectively shaping the tumor’s adaptive capacity. Although this four-niche framework has been described across diverse cancers, including pancreatic, breast, and brain tumors, the composition and metabolic features of each niche are highly variable across tumor types and even within individual tumors. For example, while the perivascular niche is often associated with proliferative cells, it can alternatively be enriched for stem-like populations with distinct metabolic programs depending on tumor context. Likewise, the functional roles assigned to each niche, such as invasion at the tumor edge, are primarily derived from specific model systems and may not be universally applicable.

Importantly, identical oncogenic drivers can be routed into different metabolic niches depending on local oxygen and nutrient conditions. For instance, *KRAS*^*G12D*^/*TP53*-mutant cells may engage branched-chain amino acid oxidation in well-oxygenated NSCLC but suppress this pathway in the more hypoxic setting of PDAC [41]. This context-dependent metabolic routing highlights why static, single-time-point biopsy analyses often fail to capture the full spectrum of tumor metabolic dependencies and adaptive states.

### Temporal metabolic heterogeneity

**Fig. 4:**
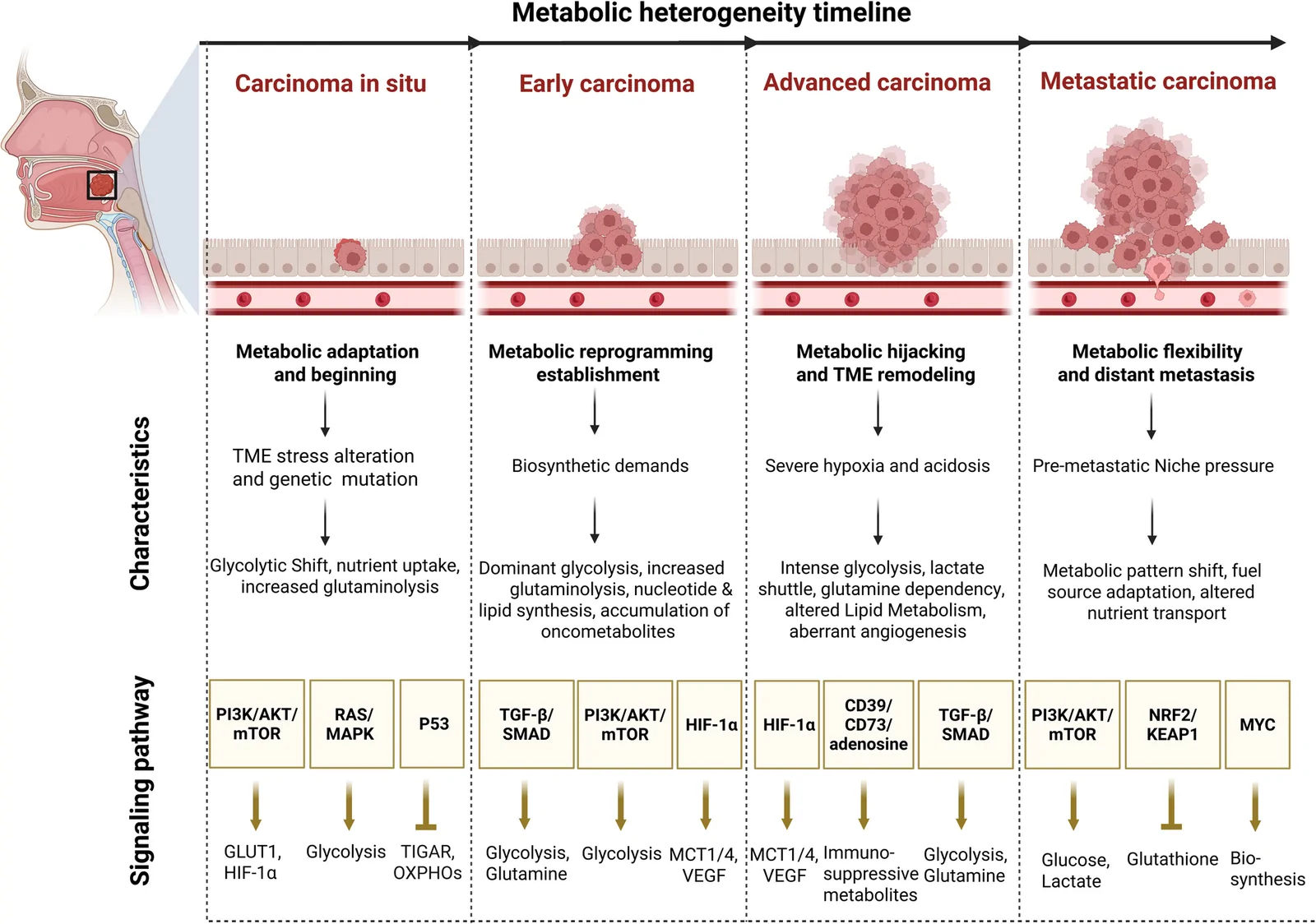
Dynamic evolution of metabolic heterogeneity across the stages of cancer progression. The schematic illustrates the stage-specific reprogramming of tumor metabolism and associated signaling pathways during cancer progression. In cancer in situ, cancer cells undergo an initial adaptive glycolytic shift. Early cancers subsequently reconfigure metabolism to meet increased biosynthetic demands. Locally advanced cancers experience metabolic hijacking and microenvironmental remodeling driven by hypoxia, while disseminated cancer cells exhibit remarkable metabolic flexibility, utilizing alternative fuels such as lipids and lactate to support colonization of distant organs. Figure created using BioRender (https://biorender.com)

Temporal metabolic heterogeneity refers to the dynamic evolution of tumor metabolic phenotypes over time. In contrast to spatial heterogeneity, which reflects metabolic differences across distinct tumor regions at a single time point, temporal heterogeneity captures the sequential reprogramming of metabolism that occurs during oncogenesis, tumor progression, treatment, and recurrence (Fig. 4). This ability to adapt metabolically is a key driver of tumor fitness, therapy resistance, and ultimately clinical relapse. Importantly, temporal metabolic changes are not spatially uniform but are instead strongly patterned across tumor architecture. A cell’s metabolic trajectory is shaped by its initial spatial positioning, such as in perivascular or hypoxic regions, as well as by the evolving local microenvironment over time. Consequently, spatial context not only constrains baseline metabolic states but also biases how those states adapt during tumor progression.

#### Drivers of temporal metabolic shifts

Cancer metabolic phenotype is not fixed but evolves dynamically through continuous clonal selection and adaptation. Temporal metabolic heterogeneity arises from the interplay of intrinsic tumor evolution and extrinsic environmental pressures. Genomic instability generates a diverse array of subclones with distinct metabolic capabilities, and through clonal competition, those subpopulations that most efficiently exploit available nutrients and withstand local stresses, such as hypoxia, acidosis, or limited glutamine, gain a proliferative advantage and come to dominate the tumor ecosystem [110]. This evolutionary process is further accelerated by therapeutic interventions, including chemotherapy, radiotherapy, and targeted therapies, which act as selective bottlenecks, eliminating metabolically vulnerable clones while permitting expansion of resistant subclones. These surviving populations frequently adopt alternative metabolic programs, such as shifting from glycolysis to OXPHOS or increasing reliance on fatty acid oxidation, underpinning acquired therapy resistance [111].

These temporal metabolic dynamics are tightly intertwined with constantly evolving host–tumor interactions that impose shifting constraints. For example, the tumor’s own remodeling of vasculature can create cycles of hypoxia and reperfusion, continuously altering selective pressures on metabolic pathways. Systemic host factors also influence tumor metabolism. For example, cachexia alters circulating nutrient levels, forcing cancer cells to adapt their metabolic programs to survive [112]. At the same time, the host immune system exerts selective pressure by eliminating vulnerable clones and inducing metabolic immunosuppressive enzymes, such as IDO1, in surviving cells through cytokines like IFN-γ. Together, these factors illustrate that temporal metabolic heterogeneity arises from the continuous interplay between tumor genetic diversity, therapeutic selection, and dynamic host-mediated pressures, shaping both tumor survival and immune evasion [113]. These temporally acquired metabolic states often converge on distinct spatial niches within the tumor. For instance, subclones selected under chronic hypoxic stress preferentially localize to perinecrotic regions, whereas therapy-resistant, OXPHOS-dependent populations are frequently enriched in perivascular or intermediate zones.

#### Evolution in tumor progression

Temporal metabolic heterogeneity is driven by adaptive rewiring to meet stage-specific challenges [2]. During early carcinogenesis, nascent tumor cells often maintain a metabolic profile similar to their tissue of origin, relying primarily on OXPHOS to efficiently generate energy for initial, relatively slow growth [2, 114]. As the primary tumor expands and outgrows its blood supply, hypoxic regions emerge, and the dominant phenotype shifts toward aerobic glycolysis. This reprogramming, often orchestrated by HIFs, ensures rapid ATP production while generating glycolytic intermediates that serve as biosynthetic precursors for nucleotides, proteins, and lipids, supporting accelerated proliferation. Concurrently, glutaminolysis provides nitrogen and carbon for continued biomass synthesis. During metastasis, cancer cells face severe metabolic stress: CTCs must survive anoikis and oxidative stress, often through upregulation of antioxidant pathways, including enhanced glutathione synthesis and NADPH production. Successful colonization of distant organs necessitates a second phase of metabolic adaptation, tuned to the nutrient landscape of the new microenvironment. For example, in lipid-rich niches such as bone marrow or liver, disseminated cells frequently rely on fatty acid oxidation (FAO) as a primary energy source, supporting survival and outgrowth in these secondary sites [115].

#### Response to therapy

Upon therapeutic exposure, cancer cells undergo a temporal cascade of metabolic adaptations that promote therapy resistance. The initial acute response involves rapid metabolic rewiring: inhibition of a dominant pathway, such as PI3K/AKT/mTOR, can trigger compensatory increases in OXPHOS and mitochondrial dependency, allowing cells to bypass the targeted blockade [116]. A subset of surviving cells may subsequently stabilize into a therapy-tolerant persister (TTP) state, characterized by slow proliferation and a pronounced metabolic shift, often from glycolysis toward mitochondrial metabolism fueled by fatty acid oxidation or amino acids. These TTPs are maintained by activated stress-response pathways, including KEAP1/NRF2, and serve as a reservoir for eventual relapse [117]. In extreme cases, a subpopulation may enter metabolic dormancy, adopting a quiescent, low-biosynthesis phenotype that minimizes energy demands and evades therapies targeting proliferating cells. This dormant state, regulated by mechanisms such as glutamine synthetase activity and mTORC1 suppression, represents a long-term reservoir for disease recurrence and metastatic outgrowth [118, 119]. This temporal metabolic adaptation occurs in a spatially non-uniform manner and ultimately reinforces intratumoral heterogeneity. For example, OXPHOS-dependent persister cells emerging under PI3K inhibition preferentially localize to perivascular and moderately oxygenated regions, where residual mitochondrial function can be maintained [120, 121], whereas cells in the central hypoxic core remain predominantly glycolytic. As a result, a single therapeutic pressure can simultaneously select for distinct metabolic states across spatial compartments, generating a mosaic of resistance programs that is difficult to eliminate with monotherapy targeting any single metabolic pathway.

## Functional consequences of metabolic heterogeneity

Metabolic heterogeneity enables tumors to flexibly adapt to spatial, temporal, and therapeutic pressures, supporting survival under diverse and often hostile conditions. It drives region- and stage-specific metabolic programs that fuel proliferation, invasion, immune evasion, and metastatic dissemination. Importantly, this heterogeneity underlies therapy resistance, as distinct subpopulations can employ alternative pathways or enter dormant states, creating reservoirs for relapse and disease progression.

### Metabolic drivers of immune evasion

The relationship between tumor metabolism and immune evasion is often described through discrete mechanisms, including nutrient competition, immunosuppressive metabolite production, checkpoint regulation, and recruitment of suppressive immune cells. However, these processes are highly interconnected and frequently redundant, and can even show context-dependent dual effects. For example, lactate suppresses T-cell function through acidification and promotes MDSC recruitment via HCAR1 signaling, yet can also serve as an alternative fuel for certain T-cell subsets under stress [122, 123]. Causality is also bidirectional, as inflammatory signals can reprogram tumor metabolism. For example, IFN-γ–induced IDO1 links immune activation to tryptophan metabolism, creating self-reinforcing feedback loops [113]. Accordingly, tumor–immune metabolic interactions should be viewed as an integrated network in which compensatory pathways limit the efficacy of single-target interventions. Clinically, this redundancy is reflected in the failure of the IDO1 inhibitor epacadostat combined with pembrolizumab in the ECHO-301 melanoma trial, which showed no improvement in progression-free survival despite strong preclinical rationale [124].

#### Nutrient competition

Subpopulations of cancer cells display distinct nutrient preferences, generating compartmentalized zones of nutrient depletion that selectively impair local immune cell function [125, 126]. Glycolysis-dependent tumor cells are frequently localized in hypoxic regions [90] and, together with the disordered architecture of tumor vasculature, exacerbate glucose scarcity in the tumor core, intensifying metabolic stress. Effector T cells rely heavily on glycolysis to meet energetic and biosynthetic demands; competition with tumor cells for glucose compromises T cell function, resulting in reduced proliferation, diminished secretion of key cytokines such as IFN-γ, and impaired cytotoxic activity, ultimately undermining anti-tumor immunity [127, 128]. In contrast, resting NK cells predominantly depend on OXPHOS to maintain basal energy homeostasis. Upon activation via cytokines (e.g., IL-2, IL-15) or engagement of activating receptors (e.g., NKG2D, DNAM-1) by tumor ligands, NK cells rapidly shift toward glycolysis to meet increased energetic and biosynthetic requirements. Although glucose limitation can impair NK cell effector functions, NK cells exhibit metabolic plasticity, utilizing alternative substrates such as fatty acids or glutamine to sustain survival and, in certain contexts, preserve or enhance cytotoxic potential. Nevertheless, prolonged or severe glucose deprivation can drive functional exhaustion or apoptosis. This dynamic adaptability underscores the metabolic flexibility of NK cells as a critical determinant of their efficacy in cancer immunotherapy [129].

Amino acids are essential for immune cells, serving as building blocks for antibodies and cytokines, supporting proliferation, regulating metabolism, and mediating activation signals. Key amino acids such as glutamine, arginine, and tryptophan are critical for T cells, macrophages, and overall immune function. Tumors can impair immunity through multiple strategies, including competitive nutrient uptake. Cancer cells often overexpress amino acid transporters, such as SLC7A5, to outcompete neighboring immune cells [130], and SLC1A5 (ASCT2) to avidly consume glutamine, creating a glutamine-depleted environment that compromises T cell activation and effector function [131].

Beyond uptake, cancer cells express catabolic enzymes that actively degrade specific amino acids. Subpopulations overexpressing IDO1 deplete tryptophan while generating kynurenine, whereas others with high arginase expression deplete arginine, creating a spatially heterogeneous amino acid-deprived TME [132]. Tryptophan starvation inhibits T cell cycle progression, and kynurenine acts as an aryl hydrocarbon receptor (AhR) ligand, promoting regulatory T cell differentiation and T cell exhaustion. Arginine depletion impairs T cell receptor signaling and downregulates CD3ζ expression, rendering T cells unresponsive [133]. In addition, tumors sequester exogenous fatty acids and cholesterol via scavenger receptors such as CD36 and SR-B1 [134], depriving T cells of lipids required for membrane synthesis and signaling, thereby impairing proliferation and function. This nutrient restriction also compromises dendritic cell antigen presentation, further diminishing T cell activation and anti-tumor immunity.

#### Secretion of immunosuppressive metabolites

Metabolic byproducts of cancer cells can exert paracrine effects on immune cells, establishing a broadly immunosuppressive field (Table 2). Glycolytic tumor subpopulations export high levels of lactate, creating extracellular acidosis that suppresses the cytolytic activity of T cells and NK cells [135]. Beyond its acidic effects, lactate acts as a signaling molecule. For instance, lactate can activate the HCAR1 receptor in colorectal tumor cells, inducing chemokines CCL2 and CCL7, which recruit immunosuppressive CCR2⁺ polymorphonuclear- MDSCs to the TME. Ablation of HCAR1 in murine colorectal tumors reduces CCR2⁺ PMN-MDSC infiltration and enhances CD8⁺ T cell activation, indicating an indirect regulatory effect of lactate on T cells [123]. Lactate also promotes histone lactylation, upregulating NUPR1 in macrophages and perpetuating a feedback loop that reinforces immunosuppression [136]. Additionally, lactate drives polarization of TAMs toward an M2-like, pro-tumor phenotype and enhances the suppressive function of regulatory T cells [137]. The roles of lactate and its downstream lysine lactylation in tumor immune suppression and microenvironmental remodeling have been comprehensively reviewed [65, 138]. These studies highlight lactylation as an important epigenetic link between metabolic reprogramming and coordinated immunosuppression across multiple immune cell populations. Other tumor-derived metabolites contribute to immune evasion: adenosine, generated by CD39/CD73 on tumor or immune cells, binds A2A receptors on T cells, NK cells, and dendritic cells, directly inhibiting proliferation, cytotoxicity, and cytokine production [139, 140]. Carbon monoxide produced by cancer cells similarly suppresses T cell proliferation and effector function [141]. Intriguingly, emerging evidence suggests that mitochondrial RNA modifications act as important regulators of metabolic reprogramming and immune cell fate within the TME, linking epitranscriptomic regulation to immunotherapy responses [142].

**Table 2:** Metabolic drivers of tumor immune evasion

Metabolic feature	Key metabolites	Target immune cells	Outcome
Aerobic glycolysis	Lactate, H⁺ ions	T cells, NK cells, macrophages	Contribute to T/NK cell dysfunction and M2 macrophage polarization [143]
Glutamine metabolism	Ammonia, α-KG	T cells, macrophages	Impair T cell impairment and M2 macrophage polarization [131]
Tryptophan metabolism	Kynurenine (IDO1/TDO)	T cells, DCs	Associated with T cell exhaustion, Treg induction and DC tolerization [144]
Adenosine generation	Adenosine (via CD39/CD73)	T cells, NK cells, Tregs, MDSCs	Promote T/NK cell dysfunction and recruitment of Tregs/MDSCs [145,146]
Mitochondrial dysfunction	ROS, succinate	Macrophages, T cells	Link to M2 macrophage polarization and T cell dysfunction [147]
Lipid metabolism	Oxidized lipids, prostaglandins	DCs, T cells, MDSCs	Contribute to DC dysfunction, T cell exhaustion and MDSC expansion [148,149]

#### Metabolic regulation of immune checkpoints

In the TME, immune checkpoints, normally protective regulatory mechanisms, are co-opted by cancer cells to drive T cell exhaustion and facilitate immune evasion. Subpopulations of cancer cells with distinct metabolic phenotypes can modulate the expression of immune checkpoints, such as PD-L1 and CTLA-4, thereby suppressing immune cell function through diverse signaling pathways (Table 3). The metabolic heterogeneity among tumor subgroups gives rise to distinct mechanisms of immune checkpoint regulation, highlighting the need for future therapeutic strategies that employ “combined strikes” targeting multiple metabolic compartments simultaneously.

**Table 3:** Influence of differential metabolic conditions on immune ligand expression

Metabolic microenvironment	Main signals	Immune ligands	Function
Hypoxic area	HIF-1α	PD-L1, CD47, PVR (CD155)	Enhanced PD-L1 expression suppresses T cell and NK cell–mediated antitumor responses, while upregulation of CD47 promotes immune evasion by inhibiting macrophage-mediated phagocytic clearance [150–152].
Perivascular area	Inflammatory signals (IFN-γ), AKT/mTOR pathway	PD-L1, MHC-1	IFN-γ signaling can induce PD-L1 upregulation, and in certain contexts may also be associated with MHC-I downregulation, thereby contributing to tumor immune evasion [150,153,154].
High acidic area	Low PH, lactate	PD-L1, GPR81 receptor	Lactate induces PD-L1 expression and activates GPR81 on immune cells to transmit inhibitory signals [155,156].
Metabolite-rich area	Specific metabolite accumulation	PD-L1, VEGF	Succinic acid stabilizes HIF-1α, leading to PD-L1 upregulation and enhanced angiogenesis [157–159].
Invasive area	NF-κB	PD-L1, NKG2D ligands (MICA/B)	NF-κB activation upregulates PD-L1 and induces shedding of MICA/B, impairing NK-cell recognition [160,161].
Intermediate area	Oncogene (MYC), adenosine	PD-L1, CD73	MYC directly drives PD-L1 transcription and indirectly upregulates CD73, potentially enhancing adenosine-mediated immunosuppression [162,163].
Necrotic area	ATP, HMGB1	CD39/CD73, PD-L1	Released ATP is converted to adenosine by CD39/CD73, while death-associated inflammation indirectly induces PD-L1 expression [164–166].
Nutrient-depleted area (glutamine and tryptophan)	ER stress, GCN2	PD-L1, IDO1	Endoplasmic reticulum stress upregulated PD-L1; tryptophan depletion activates IDO1 expression, and its product kynurenine may further suppress T cells and promote Treg generation [167–170].

#### Metabolic reshaping of the immunosuppressive microenvironment

Metabolically heterogeneous cancer cells also selectively recruit and sustain immunosuppressive populations. Lipid-rich tumor subpopulations release oxidized lipids and cytokines that promote the expansion and activation of MDSCs, which potently suppress T cell function and exhibit high oxidative metabolism, further depleting local amino acids such as cysteine and arginine. Similarly, lactate accumulation and hypoxia drive dendritic cell dysfunction, impairing antigen presentation and T cell priming [171]. In contrast to effector T cells, the metabolic landscape of the TME favors Tregs, which in turn efficiently utilize low glucose and high lactate, allowing them to thrive in immune-hostile niches maintained by tumor metabolic activity [137, 172]. This metabolic–immune crosstalk underlies the three canonical immune phenotypes: inflamed, excluded, and desert, by shaping the extent, localization, and functional persistence of immune cell infiltration within TME [173].

### Metabolism and treatment resistance

Resistance to therapy remains a major challenge in oncology, arising from a dynamic interplay between tumor-intrinsic adaptability and microenvironmental influences. This complex process is fundamentally driven by intratumoral heterogeneity, which generates subclonal populations with diverse genetic, epigenetic, and metabolic states, providing a reservoir for treatment evasion and disease progression.

Under therapeutic pressure, a subset of cancer cells can enter a reversible, non-mutational DTP state. These cells are characterized by quiescence, extensive epigenetic remodeling, and metabolic rewiring, often toward oxidative metabolism, and activate programs linked to stemness and EMT, rendering them resistant to therapies targeting proliferating cells and serving as a reservoir for long-term relapse and clonal evolution [174, 175]. Concurrently, cancer cells exhibit remarkable signaling plasticity. Inhibition of a primary oncogenic pathway, such as EGFR or PI3K, frequently triggers compensatory activation of bypass routes. For example, PI3K inhibition can induce feedback reactivation of ERK or mTOR signaling [176], whereas EGFR blockade often upregulates alternative receptors like AXL or MET, sustaining proliferative signaling and promoting EMT [177]. These adaptive signaling events are further reinforced by direct genetic evolution, including secondary mutations in drug targets (e.g., EGFR T790M) or amplification of bypass pathways, cementing therapeutic resistance.

The TME acts as both instructor and sanctuary. Stromal cells, including CAFs and immune cells, secrete cytokines such as IL-6 and TGF-β, activating pro-survival pathways in cancer cells [175]. Cancer-associated adipocytes supply fatty acids that shift tumor metabolism toward β-oxidation, enhancing survival under stress [178]. Physical stressors such as hypoxia further reinforce tolerance by stabilizing HIF signaling, upregulating drug efflux pumps, and fostering an immunosuppressive niche. Hypoxia not only induces metabolic rewiring but also reinforces cancer stemness and therapeutic resistance through interconnected signaling pathways, forming a self-sustaining “vicious triad” that promotes persistent treatment failure [179]. These interconnected adaptive strategies, persister phenotypes, signaling feedback, metabolic rewiring, and stromal support, form a robust defensive network. This highlights the clinical imperative for rational combination strategies, including co-targeting primary drivers and compensatory pathways (e.g., EGFR + MET inhibitors) or combining pathway inhibitors with agents that disrupt the epigenetic or metabolic dependencies of persister cells. Early detection of these adaptive states through molecular and metabolic biomarkers will be essential for timely intervention and prevention of relapse.

Collectively, these interconnected escape mechanisms are not merely theoretical, as reflected in the inconsistent clinical performance of metabolic therapies. Glycolytic inhibitors have largely stalled at early clinical development, metformin shows context-dependent efficacy (beneficial in head and neck cancer but largely neutral in lung cancer), and single-node targeting strategies, such as glutaminase inhibition without concurrent mTOR blockade, often fail due to rapid adaptive resistance. These observations underscore a central principle: metabolic plasticity is both spatially and temporally organized. Effective therapeutic strategies must therefore anticipate emergent adaptive states rather than target only the current metabolic configuration. Accordingly, combination regimens that pair primary metabolic inhibition with blockade of compensatory pathways, ideally informed by real-time metabolic imaging or liquid biopsy monitoring, may offer a more robust approach to overcoming tumor adaptability.

## Therapeutic implications

Metabolic heterogeneity within tumors drives immune evasion, therapy resistance, and differential susceptibility to treatment, creating distinct metabolic niches that support persister cells and immunosuppressive populations. Targeting these diverse metabolic states through combination strategies, such as co-inhibiting primary oncogenic pathways, compensatory signaling, and metabolic dependencies, offers a promising avenue to enhance therapeutic efficacy and prevent relapse.

### Targeting metabolic vulnerabilities

Despite abundant preclinical evidence, translation of metabolic targeting into clinical practice has been slow. While glycolysis, OXPHOS, and amino acid metabolism represent key metabolic vulnerabilities, most inhibitors remain in preclinical development or early-phase trials (Table 4). For example, targeting glycolytic enzymes such as LDHA, PKM2, or HK2 has shown efficacy in murine models, yet no glycolytic inhibitor has advanced beyond phase I, largely due to on-target toxicity and compensatory metabolic plasticity [180]. In contrast, IDH inhibitors have achieved clinical approval, illustrating that effective metabolic targeting is feasible when the enzyme is a tumor-specific mutant rather than a broadly essential metabolic node. The success of IDH inhibitors highlights how genetically encoded oncometabolite production can create a tumor-restricted therapeutic window. However, most cancers lack such singular dependencies. Instead, oncometabolite-like effects often arise from the local accumulation of common metabolites such as lactate or succinate, whose contributions to tumor progression and therapy resistance are context dependent. Addressing these more diffuse metabolic drivers will likely require combination strategies that also target their downstream epigenetic and signaling consequences.

**Table 4:** Clinical development status of metabolic targeting strategies in cancer

Target	Agents	Clinical stage	Key status	Clinical trials
IDH1	Ivosidenib, Vorasidenib	Phase II ongoing	Approved for IDH1-mutant cholangiocarcinoma, advanced solid tumors, lymphoma, diffuse glioma	NCT07260175 NCT04195555 NCT07240662
IDH2	Enasidenib,	Phase I/II completed or ongoing	Approved for IDH2-mutant advanced solid tumors, angioimmunoblastic T-cell lymphoma, myeloid neoplasms, malignant sinonasal and skull base tumors	NCT02273739 NCT04522895 NCT06176989
Glutaminase (GLS1)	Telaglenastat (CB-839)	Phase I/II completed or ongoing	Combined with cabozantinib in metastatic renal cell carcinoma; combined with chemoradiation in advanced cervical cancer; combined with talazoparib in solid tumors.	NCT03428217 NCT05521997 NCT03875313
Glutamine	DRP-104	Phase I/II ongoing or terminated	Approved for NFE2L2/KEAP1-altered NSCLC; Combined with durvalumab in advanced stage fibrolamellar carcinoma; terminated in advanced solid tumors	NCT07249372 NCT06027086 NCT04471415
OXPHOS (Complex I)	IACS-010759	Phase I completed or terminated	Completed in advanced cancers; terminated due to dose-limiting neurotoxicity and lactic acidosis, narrow therapeutic window in AML	NCT03291938 NCT02882321
Lactate transport (MCT1)	AZD3965	Phase I completed	Well tolerated; demonstrated on-target metabolic activity.	NCT01791595
Adenosine pathway (CD73)	Oleclumab (MEDI9447)	Phase I completed; Phase II congoing	Completed with MEDI4736 in advanced solid cancer; combined with anti-PD-L1 (durvalumab) in recurrent, refractory, or metastatic sarcoma.	NCT02503774 NCT03736473 NCT04668300
Arginase	CB-1158 (INCB001158)	Phase I completed	Monotherapy or with anti-PD-1, highest response rates were observed in HNSCC combined with pembrolizumab.	NCT02903914
CD36	PLT012	Phase I recruiting	Approved for solid tumors	NCT07337525
Fatty Acid Synthase (FASN)	TVB-2640	Phase I/II completed or ongoing	Completed in solid tumor; combined with bevacizumab in first relapse of high-grade astrocytoma; approved in metastatic castration-resistant prostate cancer	NCT02223247 NCT05743621 NCT03032484
IDO1	BMS-986,205	Phase I/II terminated or ongoing	Combined with nivolumab in liver cancer; combined with nivolumab in stage II-IV HNSCC; combined with nivolumab and radiation therapy with or without temozolomide in newly diagnosed glioblastoma	NCT03695250 NCT03854032 NCT04047706
PI3K/mTOR	Copanlisib (BAY80-694 6), Ipatasertib	Phase I completed	Completed in advanced solid tumor and non-Hodgkin lymphoma; completed in locally advanced or metastatic solid tumors.	NCT02253420 NCT00962611 NCT04341259

Contrary to the traditional view that tumors rely solely on glycolysis, certain cancer cell populations, particularly metastatic or therapy-resistant cells, exhibit elevated OXPHOS activity. Enhanced mitochondrial metabolism has been associated with chemotherapy resistance and poorer post-treatment survival [181]. Novel therapeutics targeting mitochondrial complex I (e.g., IACS-010759) or disrupting mitochondrial translation and biogenesis are currently under clinical investigation. In addition to inhibiting ATP production, OXPHOS targeting diminishes redox buffering and stem-like properties that support tumor recurrence. However, metabolic plasticity allows tumors to switch between glycolysis and OXPHOS under therapeutic pressure, emphasizing the need for combination approaches that concurrently target glycolytic and mitochondrial pathways or modulate redox homeostasis [182].

Amino acid metabolism represents another critical vulnerability, as rapidly proliferating tumor cells and immunosuppressive myeloid populations depend on glutamine, arginine, serine, and tryptophan for biosynthesis and signaling. Disruption of these pathways selectively impairs malignant cells, including leukemic stem cells, and can enhance antitumor immunity by relieving nutrient competition within the TME [183, 184]. Therapeutic strategies include glutaminase inhibitors, arginase blockers, and IDO/TDO inhibitors targeting the kynurenine pathway. However, clinical outcomes have been largely disappointing. The ECHO-301 trial evaluating the IDO1 inhibitor epacadostat in combination with anti-PD-1 therapy in melanoma showed no added benefit over immunotherapy alone [124], and the CANTATA trial of the glutaminase inhibitor telaglenastat in renal cell carcinoma failed to improve progression-free survival [185]. Given that amino acid dependencies vary across tumor types and immune cell populations, precise metabolic profiling is essential to guide effective, personalized interventions [180].

### Overcoming resistance linked to metabolic adaptation

Resistance to cancer therapy is frequently driven by the metabolic plasticity of tumor cells, which dynamically reprogram their metabolism under therapeutic pressure. This adaptation involves oscillating between energetic pathways, such as shifting from glycolysis to OXPHOS, activating alternative nutrient scavenging routes, and enhancing redox homeostasis to evade drug-induced cell death [135]. This metabolic plasticity in many aggressive cancers renders monotherapies targeting a single metabolic node largely ineffective. In contrast, tumors with strong oncogene-driven metabolic dependencies, such as IDH-mutant gliomas, can remain sensitive to single-agent metabolic inhibition, reflecting a more constrained and therapeutically exploitable metabolic state. For instance, simultaneously inhibiting glycolysis (e.g., with 2-deoxyglucose or HK2 inhibitors) and mitochondrial metabolism (e.g., with metformin or complex I inhibitors like IACS-010759) can block these reciprocal shifts [186–188]. However, early termination of IACS-010759 due to neurotoxicity and lactic acidosis [189] highlights that systemic dual inhibition of glycolysis and OXPHOS may narrow the therapeutic window to an unacceptable level. This underscores the need for tumor-selective delivery strategies or intermittent dosing regimens to reduce on-target toxicity in normal tissues. Notably, the extent of toxicity appears to vary among patients, reflecting differences in baseline metabolic reserve, tissue susceptibility, and overall tumor burden. Furthermore, combining metabolic inhibitors with conventional therapies may help overcome microenvironment-driven resistance. Strategies that reduce tumor acidity have been shown to enhance the activity of weak-base chemotherapeutics and improve T cell effector function. However, these effects have been demonstrated primarily in preclinical models, and their translational relevance in human tumors remains to be established [53, 190]. This combinatorial strategy also extends to exploiting metabolic dependencies induced by oncogenic signaling, although these effects remain highly context dependent. For example, the activatable prodrug Conjugate C1 has been reported to overcome multidrug resistance by targeting oncogene-driven mitochondrial dysfunction, simultaneously inhibiting drug efflux and restoring OXPHOS to promote apoptosis in selected mouse models of ovarian and lung cancer. However, whether these findings translate to human tumors with comparable genetic and metabolic contexts remains to be determined [136]. Similarly, a platinum (IV) complex combining dichloroacetate (DCA) and biotin ligands selectively disrupts mitochondrial function, impairing glycolysis and glucose oxidation to trigger apoptosis with greater potency than traditional platinum drugs in preclinical models. However, its clinical safety profile and therapeutic efficacy in humans remain to be established [137].

### Combination strategies with immunotherapy or targeted agents

Given the marked metabolic adaptability of cancer cells, enabling them to reprogram energy and biosynthetic pathways in response to therapeutic stress, combining immunotherapy with metabolic or targeted agents represents a promising strategy. However, its efficacy is expected to be highly tumor-type specific and strongly dependent on the pre-existing immune contexture as well as the dominant metabolic mechanisms of immune suppression within the different TME [191]. For example, pairing inhibitors of signaling cascades such as PI3K/Akt/mTOR or MAPK with cytotoxic or immune-targeted therapies can disrupt key survival pathways and improve treatment sensitivity. These principles have guided the development of next-generation combination regimens that integrate metabolic interventions with immunotherapy. Weng et al. demonstrated meaningful clinical benefits in patients with advanced biliary tract cancer, showing that adding immune checkpoint inhibitors (ICIs) to chemotherapy or tyrosine kinase inhibitors (TKIs) improved both progression-free and overall survival compared with chemotherapy alone [192]. This observation suggests that immunotherapy may achieve greater efficacy when combined with concurrent targeting of tumor metabolism and key signaling pathways. However, the specific contribution of metabolic reprogramming to the improved clinical outcomes remains unclear, and comparable combination strategies may not produce similar benefits in other tumor types with distinct metabolic architectures and adaptive programs.

Advances in nanotechnology further enhance precision delivery of combination therapies. Engineered nanomaterials can co-deliver immune modulators and metabolic inhibitors directly to tumors, improving specificity while minimizing systemic toxicity [193]. These technologies remain in early development, and their capacity to traverse the heterogeneous tumor vasculature and achieve spatially controlled, zone-specific drug release across distinct metabolic niches has yet to be demonstrated in human studies.

A major clinical frontier is leveraging metabolic interventions to convert immunologically “cold” immunosuppressive tumors into “hot” immuno-responsive ones by reprogramming the TME to relieve metabolic constraints on effective antitumor immunity. Preclinical studies in syngeneic mouse models provide proof-of-concept that metabolic reprogramming can enhance antitumor immunity; however, translation to patients remains challenging. Human tumors exhibit substantially greater metabolic heterogeneity, and the relative contribution of immunosuppressive metabolites such as lactate, adenosine, and kynurenine varies across cancer types and even within distinct regions of the same tumor. In addition, immune suppression arises from multiple interconnected mechanisms, including nutrient competition, accumulation of inhibitory metabolites, and maladaptive immune cell polarization. One strategy to relieve nutrient competition is inhibition of tumor glutaminolysis. For example, agents such as CB-839 can reduce tumor glutamine consumption, thereby increasing nutrient availability for tumor-infiltrating T cells and improving their metabolic fitness and effector function. In several preclinical models, this approach has shown synergy with immune checkpoint blockade (ICB), highlighting its potential therapeutic value [194]. However, clinical trials of glutaminase inhibitors in unselected solid tumors have produced only modest efficacy, highlighting the importance of patient stratification. These findings underscore the need to identify tumors that are truly glutamine-addicted, as well as to determine which immune cell subsets are most likely to benefit from glutamine-sparing strategies.

Directly targeting immunosuppressive metabolites that accumulate within TME represents another key therapeutic strategy. Inhibition of the adenosine axis through CD73/CD39 blockade, or suppression of lactate efflux via MCT1/4 inhibition, has been shown in vitro and in syngeneic models to restore T and NK cell function by alleviating metabolite-driven immune suppression. However, the clinical success of these approaches will likely depend on careful patient selection, particularly enrichment for tumors in which adenosine or lactate signaling represents a dominant immunosuppressive pathway. In tumors where multiple redundant metabolic suppressive mechanisms coexist, inhibition of a single axis may be insufficient, as exemplified by the failure of the IDO1 inhibitor epacadostat in the ECHO-301 trial. In addition, reprogramming the metabolism of innate immune cells offers an alternative route to reshape the tumor immune landscape. For example, DGAT1/2 inhibition reduces lipid droplet accumulation in dendritic cells and macrophages, enhancing antigen presentation and promoting a more pro-inflammatory immune phenotype in preclinical models [195, 196]. Whether these effects can be reproduced in human tumors remains an open question, as lipid accumulation in dendritic cells and macrophages may be governed by distinct and more complex metabolic programs in the clinical setting. Determining the extent to which these preclinical observations translate to human tumor immunobiology represents an important direction for future investigation.

### Patient stratification based on metabolic profiles

Metabolic heterogeneity represents a major challenge in cancer biology and in the development of durable therapies. Tumor subpopulations exhibit distinct metabolic dependencies, nutrient requirements, and therapeutic responses, complicating treatment strategies. To address this, recent efforts have employed multi-omics approaches, computational modeling, and high-throughput screening to stratify patients based on tumor metabolic profiles.

In breast cancer, for example, integrated metabolic and transcriptional profiling across subtypes revealed differences in growth rates, nutrient utilization, and biosynthetic activity [197]. By combining metabolomics, transcriptomics, and phenotypic data, researchers are beginning to map subtype-specific metabolic dependencies, providing a framework for precision oncology and targeted interventions. Similarly, a study developed a computational framework that integrates somatic mutation data with protein–protein interaction networks to define metabolic subtypes in pancreatic cancer [198]. This approach revealed distinct metabolic clusters with divergent genomic mutations and TME characteristics, demonstrating the utility of combining mutational networks with metabolic analyses for predictive stratification in cancer therapy. In renal cell carcinoma (RCC), an integrative analysis highlighted pronounced metabolic heterogeneity among patients [199]. By mapping transcriptomic signatures to pathway-level metabolic activities, distinct RCC subtypes were identified with differing glycolytic, TCA cycle, and redox metabolic profiles, which correlated with clinical outcomes. These subtype-specific metabolic dependencies offer opportunities for targeted therapeutic strategies, underscoring the translational potential of metabolic profiling in precision oncology.

## Clinical translation: diagnostic and prognostic tools

**Fig. 5:**
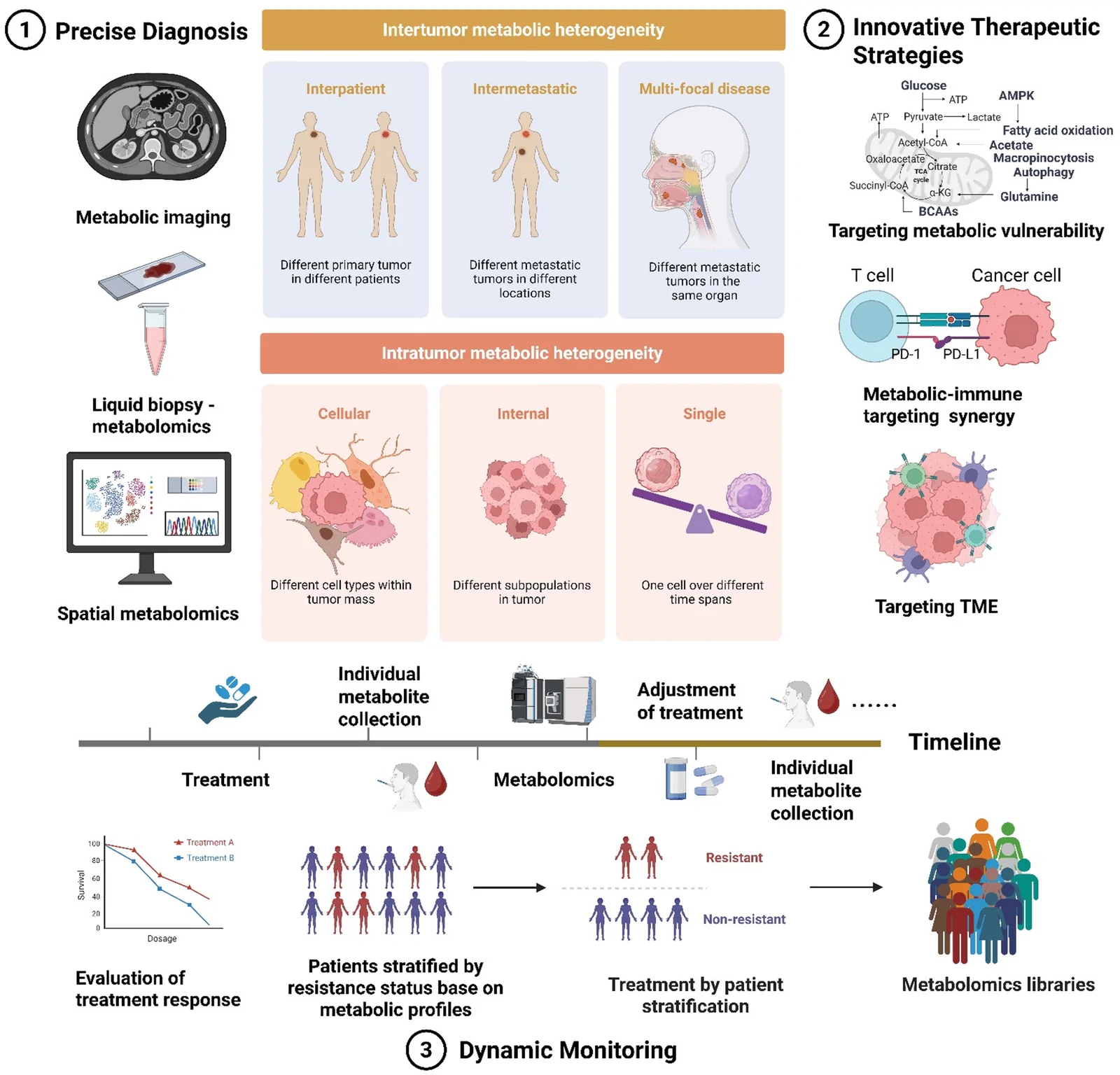
Leveraging tumor metabolic heterogeneity in clinical practice. The schematic illustrates how insights into tumor metabolic heterogeneity can be translated into tangible advances in patient care. A comprehensive understanding of both intertumor and intratumor metabolic diversity informs three key clinical pillars: (1) precision diagnosis, which employs advanced metabolic imaging, liquid biopsy-based metabolomics, and spatial metabolomics to enable molecularly guided tumor stratification; (2) innovative therapy, encompassing the targeting of metabolic vulnerabilities, integration of metabolic modulators with immunotherapy, and exploitation of microenvironmental normalization; and (3) adaptive monitoring, which employs dynamic, longitudinal assessments of tumor metabolism to evaluate treatment response, uncover mechanisms of resistance, and guide real-time therapeutic adjustments tailored to patient-specific metabolic profiles. Figure created using BioRender (https://biorender.com)

Metabolic reprogramming observed in tumors constitutes a validated reservoir of therapeutically actionable targets and clinically translatable biomarkers, fueling transformative advances across clinical oncology. These advances encompass non-invasive molecular imaging for diagnosis and treatment monitoring, evidence-based prognostic stratification, and an expanding portfolio of mechanism-driven therapeutics targeting tumor-specific metabolic dependencies, thereby establishing cancer metabolism as a foundational axis of precision oncology (Fig. 5).

### Diagnosis: metabolic imaging and biomarkers

Metabolic heterogeneity provides unique opportunities for non-invasive diagnosis and tumor characterization (Table 5). Positron emission tomography (PET) using the glucose analog ¹⁸F-fluorodeoxyglucose (FDG) remains a cornerstone in oncology for staging and assessing treatment response, directly visualizing regions of high glycolytic activity [200]. However, FDG-PET primarily captures one facet of metabolism. The development of novel radiotracers is expanding the diagnostic toolkit to map intratumoral metabolic heterogeneity more comprehensively. Tracers for glutamine metabolism (e.g., ¹⁸F-(2 S,4R)-4-fluoroglutamine), fatty acid synthesis (¹¹C-acetate), and hypoxia (¹⁸F-fluoromisonidazole) can reveal distinct metabolic subregions within a tumor, providing a more complete picture of its biological behavior and potential vulnerabilities [201–203]. For instance, the co-existence of hypoxic, glycolytic, and oxidative regions within a single lesion, detectable through multi-tracer imaging, may signal a highly adaptable and aggressive tumor [204].

Liquid biopsies are evolving beyond genetic analysis to include metabolomic profiling. The metabolic footprint of a tumor, shed into the bloodstream, can serve as a diagnostic and monitoring tool. Specific metabolite panels or ratios (e.g., lactate/pyruvate, kynurenine/tryptophan) have shown promise in distinguishing cancer patients from healthy individuals, identifying cancer types, and even detecting early-stage disease [205, 206]. Serial monitoring of circulating metabolites can provide a dynamic, systemic readout of tumor burden and metabolic adaptation during therapy, potentially identifying emergent resistance before radiographic progression. Further, techniques like mass spectrometry imaging (MSI) allow direct, label-free mapping of hundreds of metabolites across a tissue section. This reveals the functional organization of the TME, identifying distinct metabolic zones such as glycolytic hypoxic cores, lipogenic invasive fronts, and nutrient exchanges with stromal cells, providing unmatched detail on intratumor heterogeneity [207]. The recent development of spatial multi-omics technologies, such as GeoMx Digital Spatial Profiling (DSP) and MALDI-MSI, now enables simultaneous mapping of metabolic pathways, immune checkpoints, and nutrient distributions within intact tumor tissue architecture. These approaches provide an integrated framework for understanding how metabolic heterogeneity is organized in space and how it contributes to immune evasion and therapeutic resistance [208].

**Table 5:** Tools and methodologies for profiling tumor metabolic heterogeneity

Tools and methodologies	Key principle	Target	Application
Metabolic gene signatures	Computational scoring of metabolic pathway activity based on RNA-seq profiles	Gene heterogeneity	Gene expression-based metabolic signatures facilitate stratification of patients into prognostic high- and low-risk categories [209]
Mass spectrometry imaging	Direct spatial mapping of metabolites in tissue sections	Spatial heterogeneity	Identifies and visualizes distinct metabolic zones in tumor microenvironments [210]
Fluorescence lifetime imaging	Autofluorescence lifetime imaging of metabolic cofactors enables quantification of glycolytic versus oxidative metabolic activity	Cellular heterogeneity	Increased heterogeneity of NAD(P)H autofluorescence correlates with elevated tumor grade and metastatic potential [211]
Liquid biopsy metabolomics	Comprehensive biofluid metabolomics for the detection of lactate, pyruvate, amino acids, and other small molecules	Tumor heterogeneity	Supports early tumor detection and longitudinal evaluation of treatment response [212]
Genome-scale metabolic modeling	Computational frameworks combining multi-omics patient data enable the prediction of metabolic flux dynamics and identification of tumor metabolic vulnerabilities	Tumor heterogeneity	Enables systematic identification of actionable drug targets in tumors [213]

### Prognosis: metabolic subtyping and immune context

Specific metabolic profile of a tumor carries significant prognostic information. For example, high levels of glycolytic enzyme expression (e.g., HK2, LDHA) or a strong FDG-PET signal are consistently associated with poor prognosis across multiple cancer types, linking aggressive metabolism to increased proliferation, metastasis, and therapy failure [214, 215]. System level analyses have led to the identification of metabolic subtypes within histologically similar cancers. In breast cancer, distinct metabolic gene-expression signatures can stratify patients into subgroups with differential survival outcomes, superior to those based on the American Joint Committee on Cancer (AJCC) stage and prediction analysis of microarray 50 (PAM50) [216]. A recent study showed that “glycolytic-cholesterol mix subtype” in liver hepatocellular carcinoma (LIHC) is linked to worse prognosis compared to a single subtype or quiescent subtype [217].

Crucially, tumor metabolism directly sculpts the immune landscape, which is a major determinant of prognosis. Tumors with high glycolytic flux and lactate production typically exhibit excluded or dysfunctional T cell infiltrates and enriched immunosuppressive populations (Tregs, M2), correlating with poor response to immunotherapy [218, 219]. Conversely, tumors with more balanced metabolic programs may maintain a more favorable immune context. Therefore, assessing metabolic heterogeneity through imaging, gene signatures, or spatial metabolomics can provide prognostic insight not only into tumor-intrinsic aggressiveness but also into the likely state of anti-tumor immunity, informing predictions about disease course and immunotherapy efficacy.

## Challenges

Although metabolic reprogramming is a hallmark of cancer, translating this knowledge into effective therapies is hindered by tumor heterogeneity, the complex interplay with the microenvironment, and limitations in current experimental and technological approaches.

### Fundamental biological and translational complexities

The inherent biological plasticity of tumors poses a major challenge for therapeutic intervention. Metabolic dependencies are highly context-dependent, varying by tumor type, disease stage, and patient-specific factors such as diet and microbiome composition, which complicates the identification of broadly applicable targets. Intratumoral heterogeneity further generates subpopulations with distinct metabolic requirements and nutrient utilization, limiting the interpretability of bulk tumor analyses. An often-overlooked challenge is distinguishing true therapy-resistant metabolic adaptations from transient, stress-induced changes, a distinction that is critical for the development of effective and durable treatments [220].

### Limitations of preclinical model systems

A major translational bottleneck is the inability of current preclinical models to accurately recapitulate the human TME. Standard genetically engineered mouse models (GEMMs) lack human tumor antigens and a fully functional human immune system, while humanized mouse models, though incorporating human immune cells, exhibit incomplete immune reconstitution, limited cross-species compatibility, and often fail to maintain key immune populations long-term. These limitations constrain our understanding of immunotherapy responses and immune–metabolic crosstalk. Next-generation “Avatar models”, which combine patient-derived xenografts (PDXs) with a matched humanized immune system, offer a promising approach to study personalized tumor–immune interactions [221]. Ongoing efforts to enhance human cytokine support, improve myeloid cell engraftment, and promote lymphoid tissue development are critical to overcoming these challenges.

### Analytical hurdles

Despite advances in methodology, studying cancer metabolism remains technically challenging. Metabolic flux analysis, often using isotope tracing, is typically performed on bulk cell populations, masking single-cell heterogeneity and being susceptible to issues such as isotope pool dilution. Most assays capture only static snapshots, making it difficult to monitor the dynamic metabolic shifts that occur during therapy or cellular stress. While multi-omics integration, including transcriptomics, proteomics, and metabolomics, offers great potential, it is computationally intensive, and combining datasets across different molecular layers to generate a coherent model of metabolic regulation remains analytically challenging and prone to inconsistent interpretations.

### Barriers to clinical translation

Translating metabolic insights into effective cancer therapies remains a formidable challenge. The metabolic plasticity of cancer cells allows them to rapidly switch between energy sources, such as from glycolysis to oxidative phosphorylation, conferring compensatory resistance to inhibitors targeting a single pathway. This adaptability often necessitates combination therapies, which can increase the risk of off-target toxicity. Moreover, designing highly specific inhibitors for metabolic enzymes without impairing essential functions in normal tissues continues to be a major hurdle in drug development. Adding to the complexity, patient-specific metabolic variation, shaped by host physiology, means that a strategy effective in one individual may fail in another, underscoring the need for personalized approaches in metabolic therapy. This challenge is exemplified by the complex I inhibitor IACS-010759, whose development was discontinued following dose-limiting neurotoxicity and metabolic acidosis in phase I trials [188]. Likewise, systemic inhibition of glycolysis is associated with significant risks of skeletal muscle and cardiac toxicity, which have so far prevented glycolytic inhibitors from progressing beyond early clinical testing [222]. These findings highlight the inherently narrow therapeutic window of metabolic inhibitors. Future clinical success will likely depend on strategies that enable tumor-selective targeting or on robust biomarkers capable of identifying tumors that are acutely dependent on the inhibited metabolic node.

## Future directions

Advancing our understanding of tumor metabolism and overcoming associated therapeutic challenges will require research approaches that account for its dynamic, spatially distinct, and patient-specific features. Advanced model systems, including refined 3D organoids, tumor-on-chip microfluidic platforms, and patient-derived “avatar” models incorporating stromal and immune components, will be essential for identifying context-dependent metabolic vulnerabilities. High-resolution metabolic imaging techniques, such as hyperpolarized MRI, next-generation PET tracers, and spatial metabolomics, are needed to enable non-invasive, longitudinal monitoring of metabolic fluxes across distinct tumor regions in vivo. Combinatorial and personalized therapeutic strategies should move beyond single metabolic nodes to simultaneously target core dependencies and adaptive compensatory pathways, while metabolite-based biomarkers will be critical for patient stratification in precision oncology frameworks. Finally, systematic investigation of immune-metabolic crosstalk represents a particularly promising frontier, as elucidating how tumor metabolism enforces immune suppression within the TME may reveal novel avenues to enhance immunotherapy efficacy.

## Data Availability

No datasets were generated or analysed during the current study.

## Abbreviations

AJCC: American Joint Committee on Cancer
AMPK: AMP-activated protein kinase
AhR: Aryl hydrocarbon receptor
α-KG: α-ketoglutarate
BCAA: Branched-chain amino acid
CAFs: Cancer-associated fibroblasts
CSCs: Cancer stem cells
CTCs: Circulating tumor cells
D-2-HG: D-2-hydroxyglutarate
DCA: Dichloroacetate
DTP: Drug- tolerant persister
EMT: Epithelial-mesenchymal transition
ECM: Extracellular matrix
FAO: Fatty acid oxidation
FH: Fumarate hydratase
FDG: ¹⁸F-fluorodeoxyglucose
GEMMs: Genetically engineered mouse models
GSH: Glutathione
GLS1: Glutaminase
GLS2: Glutaminase 2
HNSCC: Head and neck squamous cell carcinoma
HK2: Hexokinase 2
ICIs: Immune checkpoint inhibitors
LDHA: Lactate dehydrogenase A
LPA: Lysophosphatidic acid
MSI: Mass spectrometry imaging
MDSCs: Myeloid-derived suppressor cells
IDO1: Indoleamine 2,3-dioxygenase 1
LIHC: Liver hepatocellular carcinoma
NSCLC: Non-small cell lung cancer
OXPHOS: Oxidative phosphorylation
PDAC: Pancreatic ductal adenocarcinomas
PDXs: Patient-derived xenografts
PET: Positron emission tomography
PAM50: Prediction analysis of microarray 50
RCC: Renal cell carcinoma
ROS: Reactive oxygen species
SDH: Succinate dehydrogenase
TAMs: Tumor-associated macrophages
TCA: Tricarboxylic acid
TIICs: Tumor-infiltrating immune cells
TME: Tumor microenvironment
TKIs: Tyrosine kinase inhibitors
TTP: Therapy-tolerant persister
